# Respective role of membrane and nuclear estrogen receptor (ER) α in the mandible of growing mice: Implications for ERα modulation

**DOI:** 10.1002/jbmr.3434

**Published:** 2018-05-15

**Authors:** Alexia Vinel, Amelie E Coudert, Melissa Buscato, Marie‐Cécile Valera, Agnès Ostertag, John A Katzenellenbogen, Benita S Katzenellenbogen, Ariane Berdal, Sylvie Babajko, Jean‐François Arnal, Coralie Fontaine

**Affiliations:** ^1^ INSERM‐U 1048, I2MC University of Toulouse 3 Toulouse France; ^2^ Molecular Oral Pathophysiology Team Centre de Recherche des Cordeliers INSERM‐U 1138 University of Paris‐Diderot Paris France; ^3^ UMR1132, BIOSCAR University of Paris‐Diderot Paris France; ^4^ Department of Chemistry University of Illinois at Urbana‐Champaign Urbana IL USA; ^5^ Department of Molecular and Integrative Physiology University of Illinois at Urbana‐Champaign Urbana IL USA

**Keywords:** ESTROGENS, SERMS, BONE REMODELING, GENETIC ANIMAL MODELS, BONE QCT/µCT

## Abstract

Estrogens play an important role in bone growth and maturation as well as in the regulation of bone turnover in adult*s*. Although the effects of 17β‐estradiol (E2) are well documented in long bones and vertebrae, little is known regarding its action in the mandible. E2 actions could be mediated by estrogen receptor (ER) α or β. ERs act primarily as transcriptional factors through two activation functions (AFs), AF1 and AF2, but they can also elicit membrane‐initiated steroid signaling (MISS). The aim of the present study was to define ER pathways involved in E2 effects on mandibular bone. Using mice models targeting ERβ or ERα, we first show that E2 effects on mandibular bone are mediated by ERα and do not require ERβ. Second, we show that nuclear ERαAF2 is absolutely required for all the actions of E2 on mandibular bone. Third, inactivation of ERαMISS partially reduced the E2 response on bone thickness and volume, whereas there was no significant impact on bone mineral density. Altogether, these results show that both nuclear and membrane ERα are requested to mediate full estrogen effects in the mandible of growing mice. Finally, selective activation of ERαMISS is able to exert an effect on alveolar bone but not on the cortical compartment, contrary to its protective action on femoral cortical bone. To conclude, these results highlight similarities but also specificities between effects of estrogen in long bones and in the mandible that could be of interest in therapeutic approaches to treat bone mass reduction. © 2018 American Society for Bone and Mineral Research.

## Introduction

Estrogens are key factors in bone homeostasis; they play a major role in bone growth and maturation, and adult bone turnover. Although the effects of 17β‐estradiol (E2) in long bones and vertebrae have been extensively described,^1^ little is known regarding its action in oral bone. Histomorphogenesis and bone growth dynamics associated with the morphology of the mouse mandible represent a well‐established model to study the development and evolution of these complex structures.^2^, ^3^ Alveolar bone is a very specific part of maxillary bones supporting teeth, receiving mechanical strains developed during mastication and constantly challenged by the oral microbiota and ensuing inflammation. It has been shown that the local environment is a confounding factor in the control of jaw bone density. For instance, low masticatory constraints (soft diet) induce alterations in the cancellous network within the alveolar process.^4^ In addition, particular distribution of bone modeling areas observed in the fourth postnatal week in the alveolar region could be related to diet but also to hormonal changes resulting from the endocrine activity of the ovaries.^2^, ^3^ Indeed, experimental approaches revealed that ovariectomy is associated with alterations in alveolar and condylar bone of the mandible, including a decrease in mineral density,^5^, ^6^, ^7^, ^8^, ^9^, ^10^, ^11^ an effect fully prevented by E2 replacement.^10^, ^12^

Estrogen effects are mediated by estrogen receptors alpha and beta (ERα and ERβ) that belong to the nuclear receptor superfamily.^13^ Animal studies using mutant mice models deficient for either ERα or ERβ demonstrated that ERα is absolutely required for the bone‐sparing effects of E2 on long bones and vertebrae in both male and female mice,whereas ERβ exerts only a minor protective role in female and is dispensable in male mice.^14^, ^15^ In addition, a recent publication highlighted that ERα is required to maintain the microarchitecture of maxillary alveolar bone in intact female mice.^16^ However, invalidation of ERα in mice results in disturbed serum sex steroid levels, with elevated levels of ovarian‐derived testosterone and estradiol, which in turn could act on bone mass via an activation of other receptors such as the androgen receptor.^13^, ^14^, ^17^, ^18^, ^19^, ^20^ To avoid the confounding effects of high serum sex hormones, experiments on ovariectomized mice supplemented or not with E2 are required to definitively conclude on the respective roles of ERα and ERβ subfunctions on E2 effects on jaw bone, as previously studied on long bones.^18^, ^19^, ^20^

Besides the well‐recognized role of nuclear ERα, which regulates target gene transcription (genomic action) through two independent activation functions (AFs), AF1 and AF2, a subpopulation of ERα is also present at or near the plasma membrane, where it can elicit rapid, nongenomic, membrane‐initiated steroid signaling (MISS) effects.^13^, ^21^ Although ERαMISS actions are mainly characterized by short‐term changes in signal transduction pathways, activation of membrane ERα may also impact nuclear events, including ERα activity, through phosphorylation of the nuclear receptor itself and/or of its cofactors, ultimately resulting in changes in the transcriptional activity of ERs.^21^ Experimental approaches on long bone from mice selectively deficient in ERα‐AF1 (ERα‐AF1^0^ mice) or ERα‐AF2 (ERα‐AF2^0^ mice) revealed that ERα‐AF2 is necessary for the osteoprotective effects of E2 on both cortical and cancellous bone, whereas ERα‐AF1 is only necessary in the cancellous compartment.^19^, ^22^ In addition, we and others recently demonstrated that ERαMISS effects are necessary to induce full E2 osteoprotective actions in the femur using a mouse model in which ERα cannot be directed to the plasma membrane (ERα‐C451A).^20^, ^23^

In addition to natural estrogens, selective ER modulators (SERMs) are a class of drugs acting on ERα. Among them, raloxifene (RAL), lasofoxifene (LAS), and bazedoxifene (BZA) can be used to treat postmenopausal osteoporosis.^24^, ^25^, ^26^ Experimental data on ovariectomized mice demonstrated that these three SERMs had similar effects on axial bone mass but slightly different effects on the appendicular skeleton. Importantly, all these effects require a functional ERα‐AF1.^27^ Pharmacological tools were also developed to specifically activate the ERα pool localized at the plasma membrane. The estrogen–dendrimer conjugate (EDC) consists of ethinyl‐estradiol attached to a large, positively charged, nondegradable poly(amido)amine dendrimer via hydrolytically stable linkages. EDC is highly effective in stimulating non‐nuclear signaling, but inefficient in stimulating nuclear ER target gene expression because it does not enter the nucleus.^28^ Interestingly, it was demonstrated that EDC is able to prevent bone loss in the cortical but not in the cancellous compartments in femoral and vertebral bones.^29^ However, its effect on alveolar bone, that is specific of oral bones (maxilla and mandible), is unknown. More recently, pathway preferential estrogen 1 (PaPE‐1), a molecule originated from the rearrangement of E2 steroidal structure, has been characterized as a SERM that activates membrane ERα pathway, while being insufficient to maintain a nuclear activity.^30^ PaPEs exert beneficial effects in metabolic tissues (adipose tissue and liver) and in the vasculature,^30^ but they have never been studied in bone so far.

In the present work, we used genetically modified mouse models of ER loss‐of‐function (ERα^–/–^, ERβ^–/–^, ERα‐C451A, ERα‐AF2^0^ mice) to explore the role of ERs and their subfunctions on the effects of E2 on oral bone of growing mice. In addition, we evaluated the effect of selective ERαMISS activation using EDC and PaPE‐1 on mandibular bone in ovariectomized mice.

## Materials and Methods

### Mice

Throughout all protocols, female mice were housed at the animal facility of Rangueil (US06, Toulouse, France) and kept under specific pathogen free (SPF) conditions. Mice were housed in a temperature controlled room with a 12‐hour:12‐hour light‐dark cycle and maintained with access to food and water ad libitum. Mice were fed with phytoestrogen‐free irradiated rodent diet (T.2918D.12 diet until weaning and then switched to T.2916CMI.12 diet; Envigo, Huntingdon, UK). All experimental procedures involving animals were performed in accordance with the principles established by the French Ministry of Agriculture guidelines for care and use of laboratory animals and were approved by the local Ethical Committee for Animal Care. Animals were anesthetized before surgical procedures or euthanasia, using a combination of 100 mg/kg ketamine hydrochloride (Merial, Lyon, France) and 5 mg/kg xylazine (Sigma‐Aldrich, Isle d'Abeau Chesnes, France) with intraperitoneal injection. Mice were randomly distributed in the different experimental groups and were ovariectomized before puberty, at age 4 weeks, to avoid any endogenous estrogen impregnation, as described.^20^, ^23^, ^31^, ^32^ After 2 weeks recovery, they were implanted with a subcutaneous pellet delivering either vehicle, E2 (8 μg/kg/day, pellet of 0.01 mg, 60‐day release; Innovative Research of America, Sarasota, FL, USA); as described),^20^ or PaPE‐1 (pellet of 8 mg PaPE‐1 and 12 mg cholesterol^30^) or were implanted with s.c. osmotic minipumps (Alzet, Cupertino, CA, USA; model 2004, 0.25 μL/hour, 28 days) to deliver EDC (240 μg/kg/day)^33^ or an empty dendrimer, for 3 weeks. C57BL/6J mice were obtained from Charles River Laboratories (Saint Germain Nuelles, France); the ERα^–/–^, ERβ^–/–^, ERα‐AF2^0^, and ERα‐C451A mouse lines were generated at the Institut Clinique de la Souris (Illkirch‐Graffenstaden, France) as described, and littermates control were used in each experiment.^23^, ^34^, ^35^, ^36^ All mouse lines were on C57BL/6J background, except for the ERα‐C451A mouse line that was generated on C57BL/6N background (*n* = 5 mice per group).

### Bone imaging

Mouse mandibles were dissected out after euthanasia and kept in 70% ethanol. Micro–computed tomography (μCT) analyses were performed using the Skyscan 1272, a high‐resolution X‐ray microtomography system, used at 100 kV, 100 μA, pixel size 6 μm, with a 0.25‐mm aluminum filter (Bruker microCT, Kontich, Belgium), according to the manufacturer's instructions and the recent guidelines from the American Society for Bone and Mineral Research (ASBMR).^37^, ^38^ Three‐dimensional reconstruction images were respectively obtained and analyzed with NRecon and CTAn software (Skyscan). Alveolar, trabecular, and cortical bones were analyzed from transaxial sections, respectively, between the roots of the first molar, at the mandibular condyle, and at the posterior edge of the ramus. Parameters such as bone volume per tissue volume (BV/TV), trabecular separation (Tb.Sp), number (Tb.N), and thickness (Tb.Th), as well as cortical thickness (Ct.Th) were measured. Bone mineral density (BMD) was analyzed using Bruker‐microCT BMD calibration phantoms, with concentrations of calcium hydroxyapatite (CaHA) of 0.25 and 0.75 g/cm^3^ at each studied site. Morphometric measurements were performed with DataViewer64 software (Bruker microCT). Analyses were performed between the following reference points: infradental (Id); third molar's most posterior part (M3); mandibular foramen's most posterior point (MF); condylion (Cd); and gonion (Go), as described in Supplemental Fig.  1A.

### Statistical analysis

Results are reported as the mean ± SE (*n* = 5). To test the effect of treatments, Mann‐Whitney test or one‐way ANOVA followed by a Bonferroni post‐test was performed. Two‐way ANOVA was realized to test the interaction between treatment and genotype. When an interaction was observed between two parameters, the effect of treatment was studied for each genotype with the Bonferroni post hoc test. A value of *p *< 0.05 was considered statistically significant (*p < 0.05; **p < 0.01; ***p < 0.001).

## Results

### Mandibular effects of E2 on growing mice are mediated by ERα but not by ERβ

**Figure 1:**
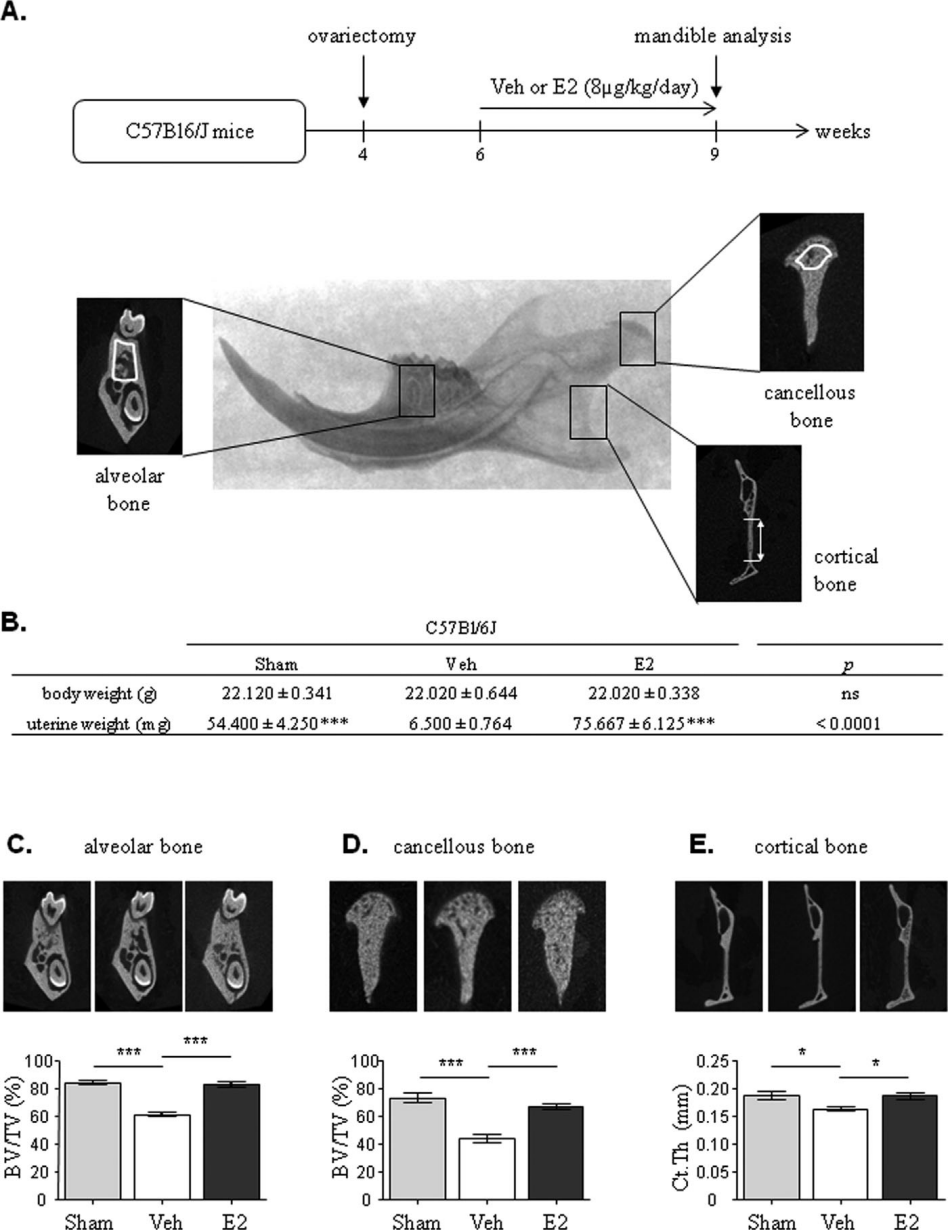
E2 treatment reverses the effects of ovariectomy on mandibular bone compartments. C57BL/6J mice were either sham operated, or ovariectomized at 4 weeks and treated with vehicle (Veh) or estradiol (E2 8 μg/kg/day) for 3 weeks. Mandibular sites analyzed: alveolar bone between first molar's roots; cortical bone at the mandible posterior edge; cancellous bone at the condyle level; the areas analyzed are delineated in white (*A*). Body and uterine weights (*B*). Representative images generated by μCT and quantification of BV/TV (*C*, *D*) or Ct.Th (*E*) of alveolar, cancellous, and cortical bones in the mandible. Results are presented as mean ± SE (*n* = 5). Veh = vehicle; BV/TV = bone volume/tissue volume; Ct.Th = cortical thickness.

In order to evaluate the effect of estrogens in our experimental conditions, and as previously described for long bone,^20^ mandibles from sham operated or ovariectomized C57BL/6J mice supplemented or not with exogenous E2 (8 μg/kg/day) were analyzed using micro‐computed tomography (μCT) in three compartments: alveolar bone between the first molar's roots, cancellous bone in the condylar area, and cortical bone in the posterior part of the mandible (Fig. 1 *A*). Prior to experiment, no significant difference was observed in body weight between the three groups. To ensure that both ovariectomy and E2 treatment were efficient, mice uteri were systematically weighted after euthanasia (Fig. 1 *B*). As expected, ovariectomy led to uterine atrophy, as reflected by a lower weight, and E2 treatment increased uterine weight (Fig. 1 *B*).

In alveolar and cancellous bones, both endogenous (ie, sham versus ovariectomized mice) and exogenous E2 led to a decrease of BV/TV (Fig. 1 *C*, *D*) and Tb.Sp with an increase of bone marrow density (BMD) (Table 1). In addition, ovariectomy was associated with a marked and significant decrease in Tb.N in cancellous but not in alveolar bone, despite a tendency in this last compartment. Exogenous E2 treatment increased this parameter in both alveolar and cancellous bones. By contrast, no effect of estrogens was observed on Tb.Th whatever the bone compartment.

**Table 1:** Changes in Alveolar and Cancellous Bone Microarchitecture Following OVX and E2 Treatment

	Sham	Vehicle	E2
Alveolar bone			
BMD (g/cm^3^)	1.204 ± 0.049***	0.786 ± 0.013	1.163 ± 0.037***
Tb.Th (mm)	0.083 ± 0.005	0.073 ± 0.002	0.075 ± 0.004
Tb.N (1/mm)	9.600 ± 0.436	8.520 ± 0.204	11.130 ± 0.588**
Tb.Sp (mm)	0.034 ± 0.003**	0.044 ± 0.001	0.029 ± 0.002***
Cancellous bone			
BMD (g/cm^3^)	1.378 ± 0.05***	0.912 ± 0.063	1.296 ± 0.015***
Tb.Th (mm)	0.038 ± 0.002	0.030 ± 0.002	0.034 ± 0.001
Tb.N (1/mm)	19.680 ± 0.370***	14.680 ± 0.255	19.870 ± 0.584***
Tb.Sp (mm)	0.032 ± 0.002***	0.062 ± 0.004	0.030 ± 0.001***

Values are mean ± SE (*n *= 5).

OVX = ovariectomy.

** *p *< 0.01 versus OVX.

*** *p* < 0.001 versus OVX.

Regarding cortical bone morphology parameters, bone thickness was significantly smaller in ovariectomized mice than in sham‐operated or E2‐treated mice (Fig. 1 *E*). Measurements between the following anatomical references: Id, M3, MF, Cd, and Go, indicated that neither ovariectomy nor E2 treatment had an effect on mandibular morphometry in these experimental conditions (Supplemental Fig.  1A, B).

**Figure 2:**
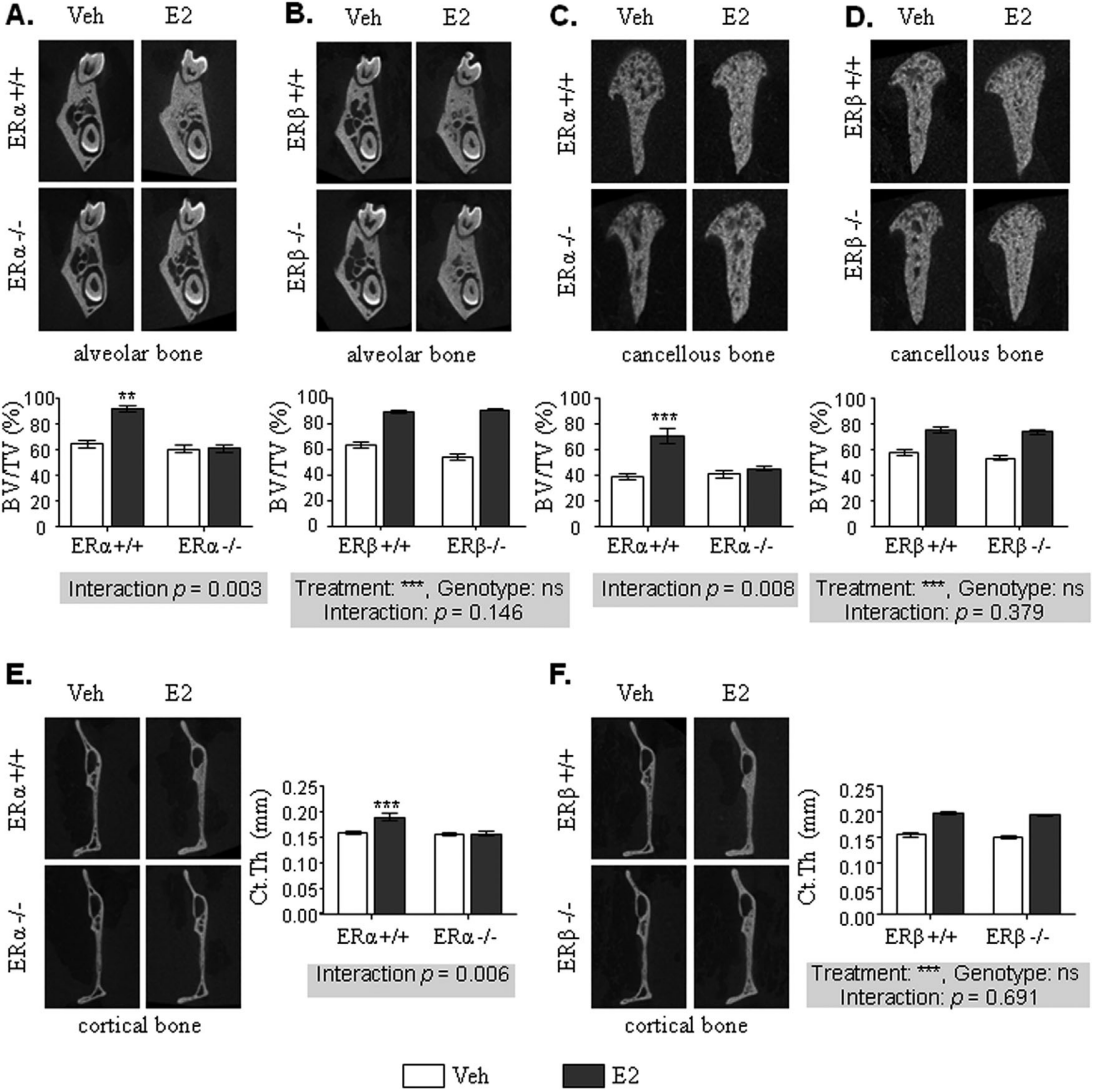
Mandibular effects of E2 are mediated by ERα but not ERβ. Four‐week‐old ERα^–/–^ and ERβ^–/–^ female mice and their littermate controls (ERα^+/+^, ERβ^+/+^) were ovariectomized and subcutaneously treated with placebo (control – white bars) or 17β‐estradiol (E2 8 μg/kg/day – dark gray bars) for 3 weeks. Mandibular bone was analyzed using μCT. Three‐dimensional representative reconstruction images and BV/TV analysis of ERα and ERβ mice alveolar (*A*, *B*) and cancellous (*C*, *D*) bones. Images and thickness measurements of ERα and ERβ deficient mice cortical bone (*E*, *F*). Results are presented as mean ± SE (*n* = 5). BV/TV = bone volume/tissue volume; Ct.Th = cortical thickness; Veh = vehicle.

Then, in order to evaluate the respective role of ERα and ERβ in E2 effects on oral bone, mandible from ovariectomized ERα^–/–^ and ERβ^–/–^ mice, and their respective wild‐type (WT) control littermates (ie, ERα^+/+^ and ERβ^+/+^, respectively), supplemented or not with E2, were analyzed. As expected, in WT mice, ovariectomy led to a complete atrophy of the uterus, whereas E2 treatment increased uterine weight. Uterine hypertrophy in response to E2 was totally absent in ERα^–/–^ mice (Supplemental Fig.  2A) and fully preserved in ERβ^–/–^ mice (Supplemental Fig.  2B). In addition and as expected, E2 treatment increased percentage of BV/TV, BMD, and Tb.N, and decreased Tb.Sp in both alveolar and cancellous mandibular bones from ERα^+/+^ mice, whereas E2 effects were totally abolished in ERα^–/–^ mice (Fig. 2 *A*, *C*; Table 2). By contrast, E2 displayed similar beneficial action in these bone compartments from ERβ^+/+^ and ERβ^–/–^ mice, as indicated by the absence of interaction between treatment and genotype (Fig. 2 *B*, *D*; Table 3). As observed in C57BL/6 mice (Table 1), trabecular thickness was not affected by either ovariectomy or E2 treatment, regardless of the genotype (Tables 2 and 3). Finally and similarly to alveolar and cancellous bone, while E2 effects on cortical thickening were absent in ERα^–/–^ mice, they remained unaffected in ERβ^–/–^ mice (Fig. 2 *E*, *F*). Altogether, these results demonstrated that the E2 effects on mandibular bone are mediated by ERα and do not require ERβ.

**Table 2:** µCT Analysis of Alveolar and Cancellous Mandibular Bone in ERα–/– Mice

	ERα^+/+^		ERα^−/−^				
	Vehicle	E2	Vehicle	E2	Interaction	Genotype	Treatment
Alveolar bone							
BMD (g/cm^3^)	0.768 ± 0.047	1.185 ± 0.089***	0.754 ± 0.029	0.771 ± 0.023	*p *= 0.002	–	–
Tb.Th (mm)	0.044 ± 0.001	0.040 ± 0.001	0.044 ± 0.003	0.043 ± 0.001	ns	ns	ns
Tb.N (1/mm)	10.252 ± 0.659	16.936 ± 1.735***	10.033 ± 0.985	9.201 ± 0.280	*p *= 0.003	–	–
Tb.Sp (mm)	0.150 ± 0.008	0.060 ± 0.023***	0.149 ± 0.01	0.155 ± 0.004	*p *= 0.002	–	–
Cancellous bone							
BMD (g/cm^3^)	0.885 ± 0.22	1.318 ± 0.065***	0.913 ± 0.037	1.048 ± 0.023	*p *= 0.002	–	–
Tb.Th (mm)	0.041 ± 0.009	0.047 ± 0.001	0.043 ± 0.002	0.044 ± 0.001	ns	ns	ns
Tb.N (1/mm)	13.085 ± 0.488	16.813 ± 0.747***	12.019 ± 0.442	13.527 ± 0.304	*p *= 0.048	–	–
Tb.Sp (mm)	0.057 ± 0.002	0.024 ± 0.001***	0.065 ± 0.005	0.056 ± 0.004	*p *= 0.004	–	–

Values are mean ± SE (*n *= 5).

ns = nonsignificant; BMD* *= bone mineral density; Tb.Th* *= trabecular thickness; Tb.N* *= trabecular number; Tb.Sp* *= trabecular separation.

*** *p *< 0.001 versus Vehicle of the same genotype.

**Table 3:** µCT Analysis of Alveolar and Cancellous Mandibular Bone in ERβ‐/‐ Mice

	ERβ^+/+^		ERβ^–/–^				
	Vehicle	E2	Vehicle	E2	Interaction	Genotype	Treatment
Alveolar bone							
BMD (g/cm^3^)	0.801 ± 0.032	1.252 ± 0.022	0.732 ± 0.033	1.240 ± 0.041	ns	ns	*p *< 0.0001
Tb.Th (mm)	0.081 ± 0.005	0.074 ± 0.005	0.080 ± 0.007	0.078 ± 0.007	ns	ns	ns
Tb.N (1/mm)	8.540 ± 0.416	12.243 ± 0.890	7.444 ± 0.458	12.641 ± 0.458	ns	ns	*p *< 0.0001
Tb.Sp (mm)	0.044 ± 0.003	0.025 ± 0.001	0.053 ± 0.007	0.023 ± 0.001	ns	ns	*p *< 0.0001
Cancellous bone							
BMD (g/cm^3^)	1.034 ± 0.029	1.354 ± 0.014	1.010 ± 0.027	1.328 ± 0.032	ns	ns	*p *< 0.0001
Tb.Th (mm)	0.037 ± 0.002	0.036 ± 0.003	0.035 ± 0.001	0.035 ± 0.001	ns	ns	ns
Tb.N (1/mm)	15.945 ± 0.959	21.213 ± 1.631	15.198 ± 0.332	20.821 ± 0.738	ns	ns	*p *< 0.0001
Tb.Sp (mm)	0.053 ± 0.003	0.025 ± 0.001	0.057 ± 0.003	0.026 ± 0.001	ns	ns	*p *< 0.0001

Values are mean ± SE (*n *= 5).

ns = nonsignificant; BMDs = bone mineral density; Tb.Th = trabecular thickness; Tb.N = trabecular number; Tb.Sp = trabecular separation.

### Both nuclear and membrane ERα are requested for full bone effects of E2 in the mandible of growing mice

**Figure 3:**
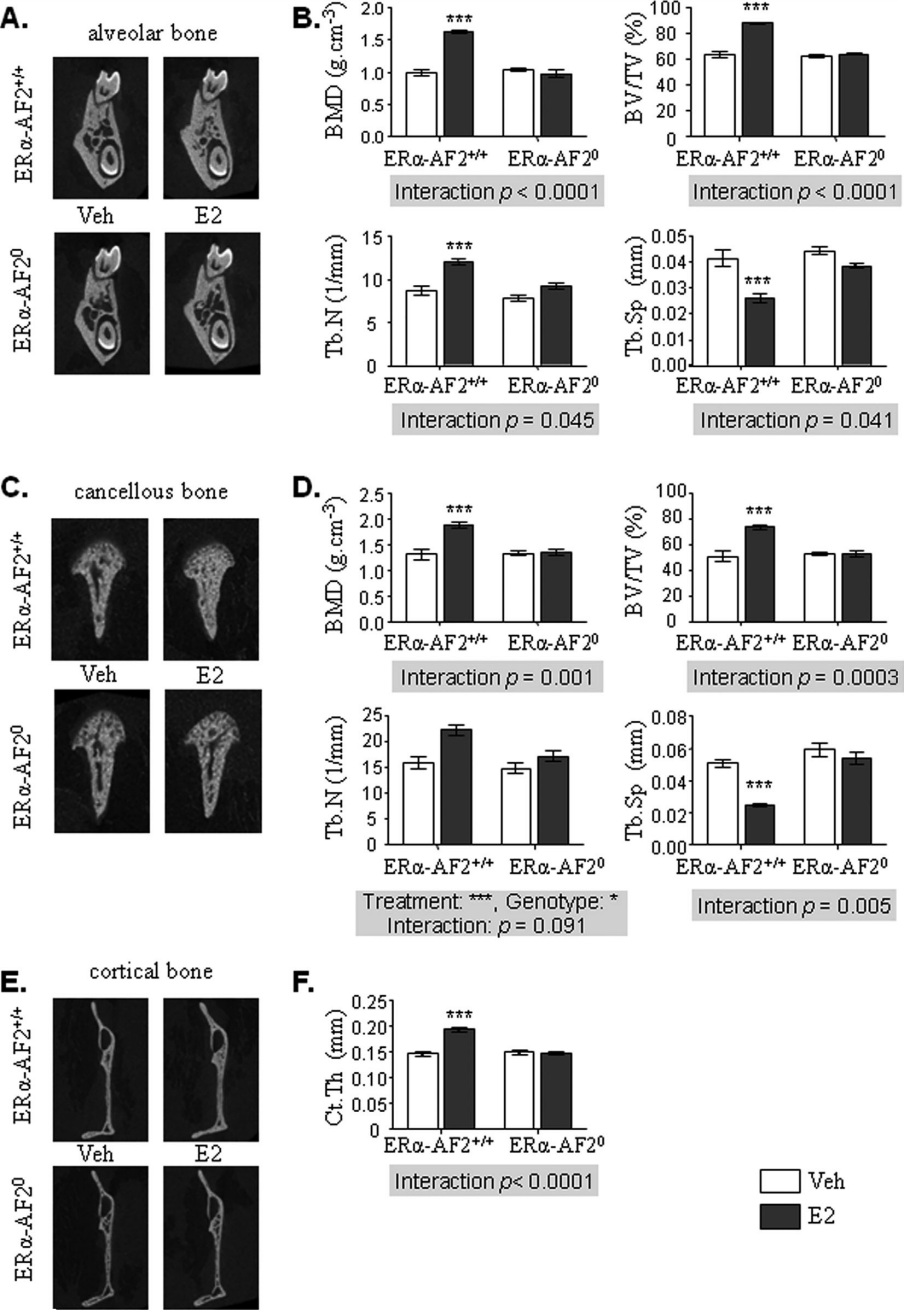
ERα nuclear pathways are necessary to mediate E2 effects on mandibular bone. Four‐week‐old ERα‐AF2^00^ and their littermate controls (ERα‐AF2^+/+^) female mice were ovariectomized and subcutaneously treated with placebo (control – white bars) or 17β‐estradiol (E2 8 μg/kg/day – dark gray bars) for 3 weeks. Mandibular bone was analyzed using μCT. Three‐dimensional representative reconstruction images of alveolar bone (*A*); and BMD, cancellous BV/TV, Tb.N, and Tb.Sp measurements (*B*). (*C*) Representative images of cancellous bone three‐dimensional reconstructions. (*D*) The same parameters as in alveolar bone were studied on condylar cancellous bone. (*E*) Indicative images of cortical bone after three‐dimensional reconstruction. (*F*) Ct.Th was measured at the posterior edge of the mandible. Results are represented as mean ± SE (*n* = 5). BMD = bone mineral density; BV/TV = bone volume/tissue volume; Tb.N = trabecular number; Tb.Sp = trabecular separation; Ct.Th = cortical thickness; Veh = vehicle.

Because both nuclear and membrane ERα are requested in E2 beneficial effects in long bone, we next explored the respective role of these ERα subfunctions in the effects of E2 on mandibular bone using ERα‐AF2^0^ and ERα‐C451A mice and their respective littermates (ie, ERα‐AF2^+/+^ and ERα‐WT, respectively). As expected, E2 increased uterine weight in WT and ERα‐C451A mice but not in ERα‐AF2^0^ mice (Supplemental Fig.  2C, D). Alveolar and cancellous bone analysis revealed that E2 effects on BMD, BV/TV, Tb.N, and Tb.Sp were completely absent in ERα‐AF2^0^ mice (Fig. 3 *A*–*D*). In addition, the increase of cortical thickness in response to E2 observed in cortical bone from ERαAF2^+/+^ mice was absent in ERα‐AF2^0^ mutant mice (Fig. 3 *E*, *F*). Altogether, as previously described in long bones and in vertebrae,^19^ these results showed that the effects of E2 on mandibular bone absolutely require ERα‐AF2.

**Figure 4:**
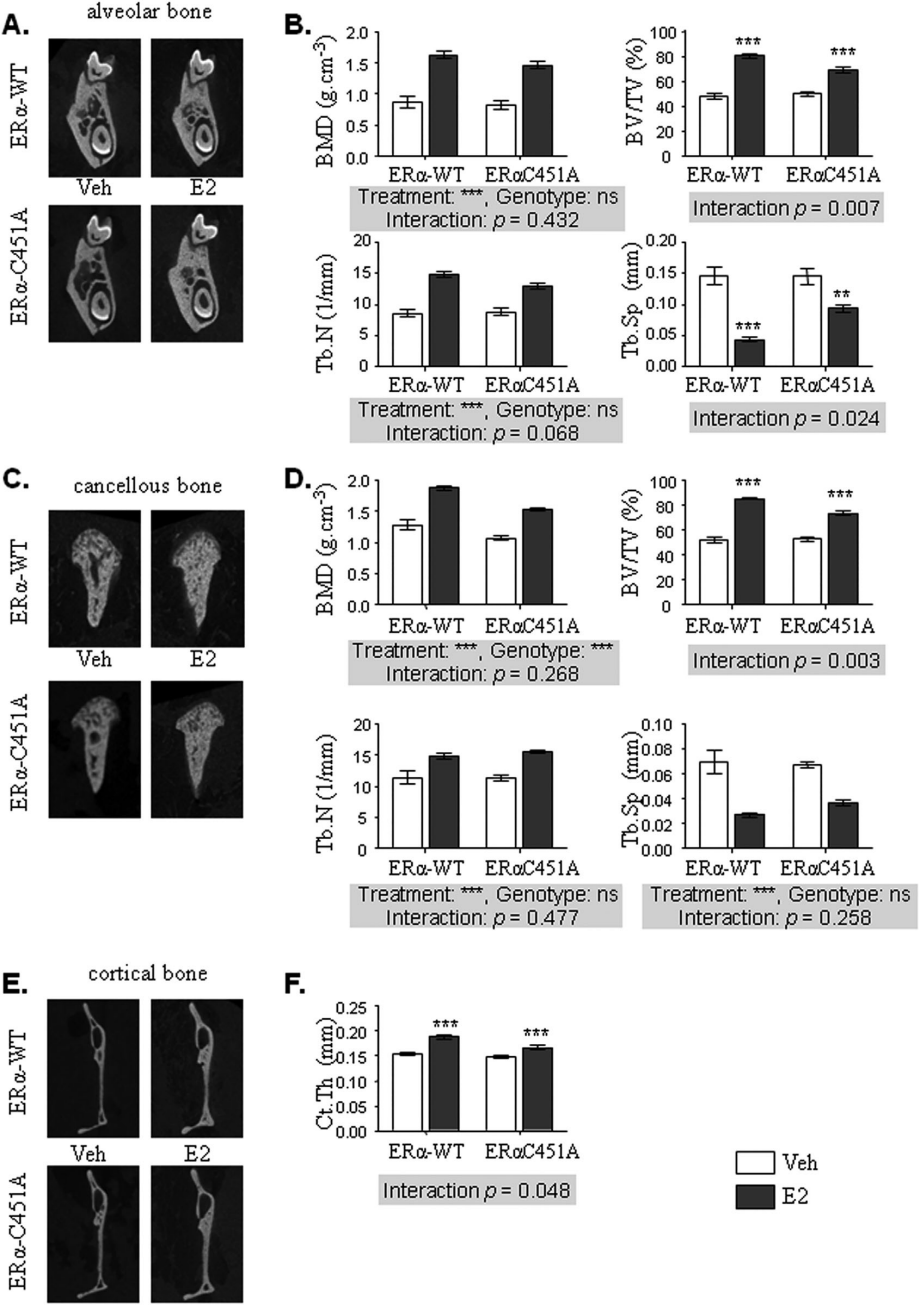
ERαMISS is requested for full E2 effects on mandibular bone. Four‐week‐old ERα‐C451A and their littermate controls (ERα‐WT) female mice were ovariectomized and subcutaneously treated with placebo (control – white bars) or 17β‐estradiol (E2 8 μg/kg/day – dark gray bars) for 3 weeks. Mandibular bone was analyzed using μCT. Three‐dimensional representative reconstruction images of alveolar bone (*A*); and BMD, cancellous BV/TV, Tb.N, and Tb.Sp measurements (*B*). (*C*) Representative images of cancellous bone three‐dimensional reconstructions. (*D*) The same parameters as in alveolar bone were studied on condylar cancellous bone. (*E*) Indicative images of cortical bone after three‐dimensional reconstruction. (*F*) Ct.Th was measured at the posterior edge of the mandible. Results are represented as mean ± SE (*n* = 5). BMD = bone mineral density; BV/TV = bone volume/tissue volume; Tb.N = trabecular number; Tb.Sp = trabecular separation; Ct.Th = cortical thickness; Veh = vehicle.

Analysis of ERα‐C451A mice revealed no difference with control mice regarding the beneficial effects of E2 on BMD and Tb.N in alveolar and cancellous bone (Fig. 4 *B*, *D*). In addition, even if E2 effects on BV/TV were still effective in both alveolar (Fig. 4 *A*, *B*) and cancellous (Fig. 4 *C*, *D*) bone from ERα‐C451A mice, they were significantly decreased compared to their control littermates in both alveolar (Fig. 4 *A*, *B*) and cancellous (Fig. 4 *C*, *D*) compartments. Tb.Sp was significantly less impacted by E2 treatment in ERα‐C451A than in control mice in the alveolar compartment (Fig. 4 *B*), but not in the cancellous compartment (Fig. 4 *D*). Finally, E2 effects on Ct.Th were significantly reduced in ERα‐C451A mice compared to WT mice (Fig. 4 *E*, *F*). All these results demonstrate that contrary to nuclear activation, ERαMISS is not absolutely required but contributes to the effects of E2 on mandibular bone.

### Selective activation of ERαMISS is sufficient to affect alveolar bone but not cancellous and cortical compartments in the mandible

**Figure 5:**
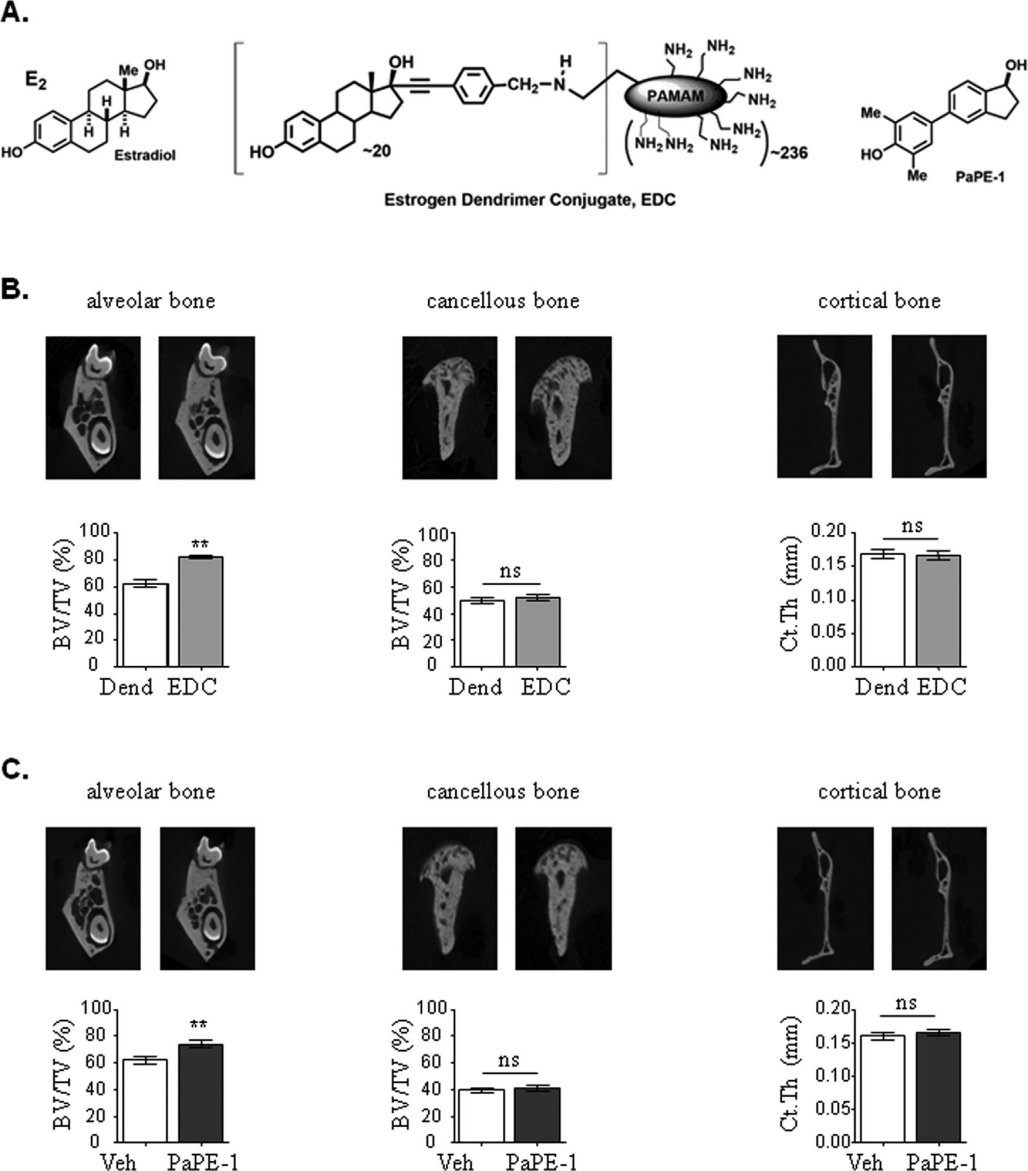
ERαMISS selective activation affects alveolar bone. Four‐week‐old C57BL/6J mice were ovariectomized and treated with either empty dendrimer (Dend) or vehicle (Veh) (control – white bars), or EDC (light gray bars), or PaPE‐1 (dark gray bars) for 3 weeks. (*A*) Chemical structure of 17β‐estradiol (E2), EDC and PaPE‐1. (*B*) Representative images generated by μCT and quantification of BV/TV of alveolar and cancellous bone, and Ct.Th from EDC‐treated mice. (*C*) Representative images generated by μCT and quantification of BV/TV of alveolar and cancellous bone, and Ct.Th from mice treated with PaPE‐1. Results are presented as mean ± SE (*n* = 5). BV/TV = bone volume/tissue volume; Ct.Th = cortical thickness; Veh = vehicle.

Because it was previously shown that selective activation of ERαMISS was able to elicit some osteoprotective effects in long bones using EDC compound,^29^ we then evaluated the impact of EDC in the mandible (Fig. 5 *A*). To this aim, C57BL/6 mice were ovariectomized and implanted with s.c. osmotic minipumps prepared to deliver vehicle or EDC. Interestingly, EDC significantly increased BMD, BV/TV, and Tb.N and decreased Tb.Sp in alveolar bone (Fig. 5 *B*, Table 4). To further extend these results, we then treated C57BL/6 mice with subcutaneous pellet of PaPE‐1, another estrogenic ligand designed to preferentially activate the membrane‐initiated signaling pathway over the nuclear‐initiated pathway of ERα. Similar to E2 and more importantly to EDC, PaPE‐1 increased BMD, BV/TV, and Tb.N whereas it decreased Tb.Sp in the alveolar compartment (Fig. 5 *C*, Table 5). By contrast, neither EDC, nor PaPE‐1 was able to affect cancellous and cortical compartments of the mandible (Fig. 5 *B*, *C*; Tables 4 and 5).

**Table 4:** µCT Analysis of Alveolar and Cancellous Mandibular Bone in C57Bl/6J Mice Following Ovariectomy and EDC Treatment

	Dend	EDC
Alveolar bone		
BMD (g/cm^3^)	0.808 ± 0.063	1.183 ± 0.018**
Tb.Th (mm)	0.069 ± 0.003	0.079 ± 0.004
Tb.N (1/mm)	9.118 ± 0.279	10.590 ± 0.556*
Tb.Sp (mm)	0.045 ± 0.002	0.037 ± 0.002*
Cancellous bone		
BMD (g/cm^3^)	1.068 ± 0.056	1.177 ± 0.029
Tb.Th (mm)	0.032 ± 0.001	0.033 ± 0.0006
Tb.N (1/mm)	15.380 ± 0.393	15.990 ± 0.455
Tb.Sp (mm)	0.059 ± 0.004	0.056 ± 0.004

Values are mean ± SE (*n *= 5).

Dend = Empty Dendrimer.

* *p *< 0.05 versus Dend.

** *p *< 0.01 versus Dend.

**Table 5:** µCT Analysis of Alveolar and Cancellous Mandibular Bone in C57Bl/6J Mice Following OVX and PaPE‐1 Treatment

	Vehicle	PaPE‐1
Alveolar bone		
BMD (g/cm^3^)	0.742 ± 0.037	0.882 ± 0.028*
Tb.Th (mm)	0.074 ± 0.002	0.081 ± 0.003
Tb.N (1/mm)	7.874 ± 0.223	8.783 ± 0.142*
Tb.Sp (mm)	0.045 ± 0.002	0.037 ± 0.043**
Cancellous bone		
BMD (g/cm^3^)	0.801 ± 0.023	0.793 ± 0.019
Tb.Th (mm)	0.039 ± 0.002	0.034 ± 0.001
Tb.N (1/mm)	14.040 ± 0.64	14.530 ± 0.34
Tb.Sp (mm)	0.066 ± 0.005	0.059 ± 0.002

Values are mean ± SE (*n *= 5).

OVX = ovariectomy.

* *p *< 0.05 versus OVX.

** *p* < 0.01 versus OVX.

## Discussion

In this study, we show that: (i) E2 effects on mandibular bone are mediated by ERα but not ERβ; (ii) these E2 effects are totally abrogated in ERα‐AF2^0^ mice and partially altered in ERα‐C451A mice; and (iii) selective activation of ERα‐MISS is sufficient to strengthen microarchitecture in alveolar but not in cancellous and cortical bone.

ERα and ERβ are the two main receptors that mediate the majority of estrogen effects.^13^ Their roles on long bones and vertebrae have been extensively explored in vivo using transgenic mouse models, that showed that E2 effects are mediated by ERα, whereas ERβ only has minor role in female long and vertebral bones and none on male bones.^14^, ^15^ However, those studies remained controversial due to several genetically engineered mice aimed at functionally ablating the ERβ, which exhibited somewhat divergent phenotypes.^14^, ^15^, ^34^, ^36^ Here, using the last generation of mouse model in which exon 3 of ERβ was completely deleted by Cre/LoxP‐mediated excision and devoid of any transcript downstream of this exon, we definitively demonstrated that the effects of E2 on mandibular bone do not require ERβ. By contrast, we showed that ERα is absolutely required because all the effects of E2 at the mandible are absent in ERα^–/–^ mice. Besides ERs, a member of the seven‐transmembrane G protein‐coupled receptor family, GPR30, has emerged as a third ER. GPR30 activation has been reported to exert several beneficial effects in the bone system.^39^, ^40^, ^41^ Thus, an interaction with GPR30 could be envisioned, but this aspect is beyond the scope of this work.

ERα acts mainly as a transcription factor in the nucleus through its AF2 function (ERα‐AF2), which allows the recruitment of coactivators.^35^ Using mouse models lacking this function (ERα‐AF2^0^), Börjesson and colleagues^19^ showed that the ERα‐AF2 function is absolutely required for E2 osteoprotective effects on cortical and cancellous bone in femur and vertebra. Here, we show that this function is also necessary to mediate the E2 action on oral bone. Besides the classical nuclear functions of ERα, this receptor is also able to mediate extranuclear signaling, which includes posttranscriptional modifications and interactions of the receptor with other molecular actors, like adaptor molecules, kinases, and G proteins.^13^ Indeed, a fraction of ERα is localized at the plasma membrane where it can elicit rapid signaling, as demonstrated in cell culture models.^42^ Based on in vitro work demonstrating that Cys‐447 of human ERα (Cys‐451 in mouse) is a crucial palmitoylation site for the receptor membrane localization,^43^, ^44^ we generated a mouse model with a point mutation of this ERα palmitoylation site (ERα‐C451A) in which membrane activation of ERα is abrogated.^23^ Using this mouse model, we recently demonstrated that the mutation leading to an abolition of membrane ERα actions also decreased E2 effects on long bone^20^; the same observation was reported by others.^45^ Here, we show that the action of E2 on mandibular bone is also altered in ERα‐C451A mice in alveolar, trabecular, and cortical compartments.

Interestingly, besides estrogens, synthetic compounds are able to bind ERα and induce specific tissue‐selective actions, being agonists and mimicking some effects of estrogens while antagonizing others.^24^, ^46^ The main explanation for the actions of these SERMs is based on the relative activation of nuclear, AF1 and AF2 ERα functions, and MISS activation.^13^ ERαMISS selective activation using EDC and/or PaPE‐1 has been shown to exert beneficial protection against vascular and metabolic disorders as well as against long‐bone demineralization without impacting sex targets.^33^ Interestingly, it has been demonstrated that selective activation of this pathway using EDC was able to exert beneficial effects on cortical but not trabecular bone of femur and vertebra.^29^ Here, we showed that both EDC and PaPE‐1 are able to counteract bone disorders induced by ovariectomy in alveolar bone but not in cancellous and cortical compartments of the mandible. These results suggest that the fine molecular mechanisms that prevail in long or vertebral bones are probably different from those that predominate in a strongly solicited bone such as the mandible, which is specifically relied to dental structure and functions.

At first glance, a pharmacological approach with EDC and PaPE‐1 on the one hand and results from the genetically modified models targeting ERα nuclear loss‐of‐function ERα‐AF2^0^ on the other hand could seem contradictory. Indeed, whereas the selective activation of ERαMISS by EDC could act on long^29^ and mandibular bone, there is a total abrogation of E2‐beneficial actions using ERα‐AF2^0^ nuclear‐deficient mice.^19^ However, it has been recently shown that nuclear ERα‐AF1 is crucial for EDC effect, highlighting the importance of the crosstalk between nuclear and extranuclear ERs to mediate beneficial effects of estrogens on long bone.^47^ In addition, it can be hypothesized that part of the action of EDC and PaPE‐1 could involve an activation of both AF1 and AF2, suggesting another level of interaction between membrane and nuclear ERα.

Estrogens are necessary not only for bone growth and maturation, but also for the regulation of bone turnover in adults. Consequently, menopause, resulting from decreased production of 17β‐estradiol (E2) due to cessation of ovarian function, is currently associated with osteoporosis, a widespread pathology characterized by bone tissue loss, leading to skeleton fragility and increased fracture risk.^48^ Although there is no consensus today, several clinical studies suggest an association between osteoporosis and oral bone loss.^49^, ^50^, ^51^, ^52^ Recent studies reported in women an association between osteoporosis and tooth loss, indicating that low femoral BMD and *T*‐score were associated with fewer remaining teeth.^53^, ^54^, ^55^ In addition, low BMD and bone stiffness were associated with low number of teeth in women but not in men.^56^, ^57^ Furthermore, studies reported that dental panoramic and measurements of mandibular cortical thickness could be used to diagnose osteoporosis and thus identify women at risk of low BMD.^58^, ^59^, ^60^ In addition, even if hormonal replacement therapy (HRT) has been extensively recognized to exert beneficial actions to prevent and treat osteoporosis, its effect on mandibular bone is still poorly described. If one observational study reported no beneficial effect of HRT in postmenopausal women on alveolar bone height and porosity reduction,^61^ several others showed that postmenopausal women receiving HRT exhibited less alveolar bone loss than untreated women.^62^, ^63^, ^64^ Interestingly, two experimental studies showed that E2 treatment in mature mice was able to reverse the effects of ovariectomy on alveolar and condylar bone microarchitecture, respectively.^10^, ^12^ Moreover, ERα has also been shown to be necessary for estrogen's protective effect on alveolar bone in intact mature mice.^16^ Here, we show that ERα is also required to mediate E2 effect at the mandible in the growing mice. In addition, it was proposed that selective activation of ERαMISS could represent an optimized alternative to induce the beneficial effects of E2 on vascular, metabolic, and long‐bone parameters^29^, ^30^, ^33^ with limited deleterious effects on breast and endometrial proliferation.^33^ The results of the present study suggest that this strategy could also offer beneficial action on mandibular bone.

Altogether, this unprecedented level of insight and dissection in the mechanisms of action of ERα in bone metabolism could be of interest to treat bone disorders and to minimize serious health risks related to such treatment (mainly venous thromboembolism and/or breast cancer). It will help to understand the mechanism of action of “old” ER modulators and to design of new ones with an optimized benefit/risk profile.

## Disclosures

All authors state that they have no conflicts of interest.

## Supporting information

Supporting Figure S1.

Supporting Figure S2.
