# Supplementary figures and images for: Respective role of membrane and nuclear estrogen receptor (ER) α in the mandible of growing mice: Implications for ERα modulation
Source: J Bone Miner Res. 2018 May 15;33(8):1520–31. doi: 10.1002/jbmr.3434 (PMC6563159; doi:10.1002/jbmr.3434)

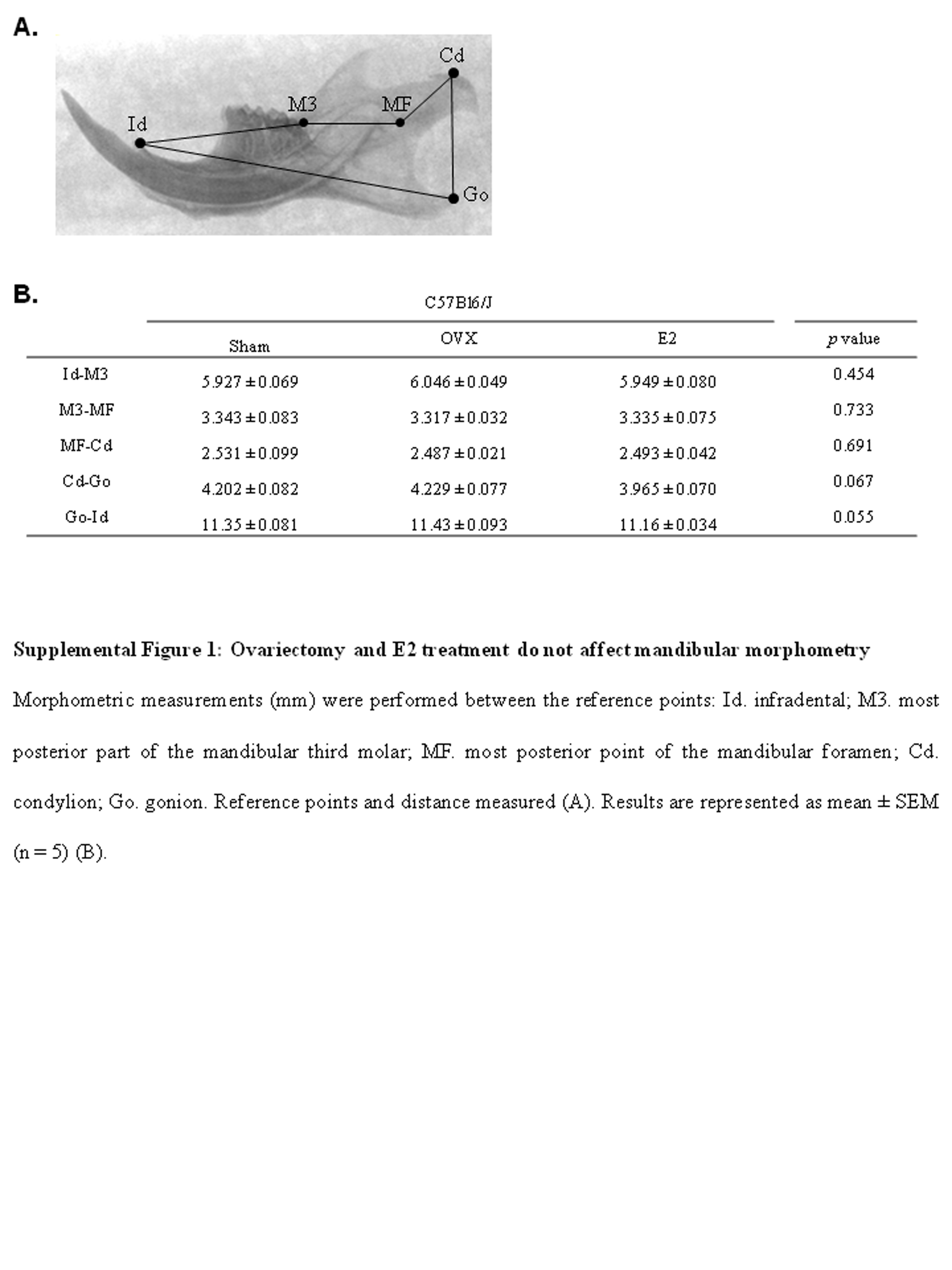

Supplement: Supplementary file 1 — Supporting Figure S1. [file JBMR-33-1520-s001.tif]

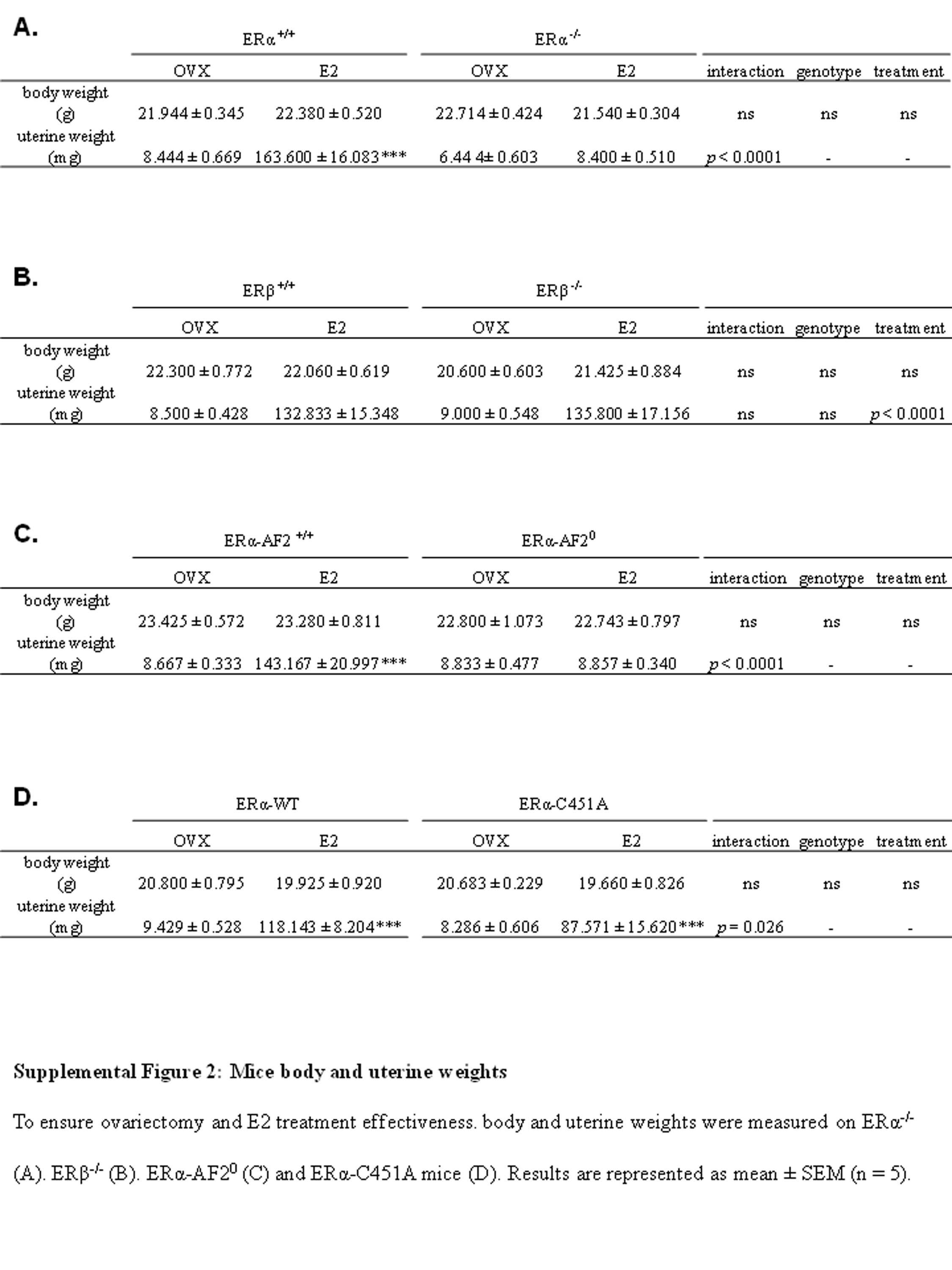

Supplement: Supplementary file 2 — Supporting Figure S2. [file JBMR-33-1520-s002.tif]
